# Ecology and Biogeography of Bacterial Communities Associated with Chloroethene-Contaminated Aquifers

**DOI:** 10.3389/fmicb.2012.00260

**Published:** 2012-07-23

**Authors:** Pierre Rossi, Noam Shani, Florian Kohler, Gwenaël Imfeld, Christof Holliger

**Affiliations:** ^1^Central Environmental Laboratory, School of Architecture, Civil and Environmental Engineering, Ecole Polytechnique Fédérale de LausanneLausanne, Switzerland; ^2^Laboratory for Environmental Biotechnology, School of Architecture, Civil and Environmental Engineering, Ecole Polytechnique Fédérale de LausanneLausanne, Switzerland; ^3^Laboratory for Ecological Systems, School of Architecture, Civil and Environmental Engineering, Ecole Polytechnique Fédérale de LausanneLausanne, Switzerland; ^4^Laboratory of Surface Hydrology and Geochemistry, University of Strasbourg/ENGEES, UMR 7517 CNRSStrasbourg, France

**Keywords:** bacterial communities, organohalide respiration, *Dehalococcoides*, chloroethenes, aquifers, T-RFLP, numerical ecology, biogeography

## Abstract

Massive usage, along with careless handling, storage, spills, and leakages made chloroethenes (CEs) one of the most abundant classes of groundwater contaminants. Anaerobic organohalide respiring bacteria (OHRB) can couple reductive dechlorination of CEs with energy conservation, a central microbial process in (enhanced) natural attenuation of CE-contaminated aquifers. Spatial variability of OHRB guild members present in contaminated sites has not yet been investigated in detail and it is not known whether the spatial localization of contaminated sites could impact differentially remediation capacities. The goal of this study was to investigate how spatially distant microbial communities responded to the presence of CEs. Bacterial communities associated with five geographically distant European CE-contaminated aquifers were analyzed with terminal restriction fragment length polymorphism. Numerical ecology tools were used to assess the separate and combined effects on the communities of their spatial localization, their local environmental conditions and their contaminant concentrations. Three spatial scales were used for the assessment of the structuration of the communities as a function of geographical distances, namely at the aquifer scale, at medium (50 km) and long (ca. 1000 km) distances between aquifers. As a result, bacterial communities were structured with an almost identical contribution by both the geographical position of the aquifer and local environmental variables, especially electron donors and acceptors. The impact of environmental factors decreased with distance between aquifers, with the concomitant increase in importance of a geographical factor. Contrastingly, CEs contributed at a low extent at the medium scale and became important only when all aquifers were considered together, at a large geographical scale, suggesting that distant communities were structured partially by a common niche specialization in organohalide respiration.

## Introduction

Invisible above ground, but apparently constantly replenished, groundwater has long been considered as an inexhaustible resource (UNESCO, 2006). Quantitative pressures leading to the depletion of groundwater reserves are nowadays coupled with the qualitative threatening of this precious element (Danielopol et al., 2003). For instance, massive usage, along with careless handling and storage, spills, and leakages have made chlorinated ethenes (CEs) one of the most abundant classes of groundwater contaminants. Numerous industries and facilities have been using CEs, notably tetrachloroethene (PCE) and trichlorethylene (TCE), as non-flammable solvents during manufacturing since the beginning of the twentieth century (Fetzner, 1998). Due to their low solubility in water, CEs can persist in the subsurface for long periods of time, causing plumes of dissolved material to remain at concentrations that are many orders of magnitude above the level of concern. CEs were initially considered as totally recalcitrant to microbial biodegradation due to their persistence in aerobic environments and on the presumption that they were not natural components of the biosphere (Bradley and Chapelle, 2010). Although abiotic processes might be involved in some of the reductive transformations observed in environmental samples, reports in the 1980s indicated that PCE and TCE were reduced biologically under anaerobic conditions into lower chlorinated ethenes, such as *cis*-dichloroethene (cDCE) and vinyl chloride (VC) and finally to the harmless ethene. Advances in this field showed that selected microorganisms were able to grow using CEs as respiratory terminal electron acceptors, a process termed “OrganoHalide Respiration” (OHR; El Fantroussi et al., 1998; Hendrickson et al., 2002; Duhamel and Edwards, 2006; Heimann et al., 2010). OHR bacteria (OHRB) have been discovered in a large variety of phyla, such as the *Firmicutes*, *Chloroflexi*, *ε-Proteobacteria*, and δ*-Proteobacteria*. These guild members, which are known to use other electron acceptors than organohalides, are referred to as *facultative* OHRB, with examples of species belonging to the *Anaeromyxobacter*, *Desulfitobacterium*, *Desulfovibrio*, *Desulfomonile*, *Geobacter*, and *Sulfurospirillum* genera (for a review see for instance Bradley and Chapelle, 2010; Smidt and de Vos, 2004). A second category of OHRB, and possibly the most intriguing, is composed of species which require an organohalide molecule as terminal electron acceptor to support their growth. Among them, *Dehalobacter* sp., has first been shown to reductively dechlorinate PCE and TCE to cDCE (Holliger et al., 1998). Currently, the highest number of *obligate* OHRB is present in the *Chloroflexi* phylum, with representatives of the “*Dehalococcoides*” genus (Tas et al., 2010). Continuing efforts are being made to evaluate reductive dechlorination processes in aquifers and to monitor *in situ* bioremediation processes, providing contrasting remediation outcomes, even under comparable environmental conditions (Bradley and Chapelle, 2010; Dowideit et al., 2010). In many systems in which OHR activities have been biostimulated by the addition of an electron donor or in which monitored natural attenuation (MNA) was conducted, incomplete dechlorination of CEs often led to the accumulation of the daughter molecules cDCE and VC, which are even more toxic than the parent compounds (Lorah and Voytek, 2004).

Fundamental biological processes resulting from local habitat conditions are being addressed nowadays both from an ecological and biogeographical point of view (Green and Bohannan, 2006; Martiny et al., 2006; Horner-Devine et al., 2007; Prosser et al., 2007). As stated by Horner-Devine et al. (2004), one of the most significant challenges in microbial ecology is to understand how microbial communities are patterned with spatial and temporal heterogeneities in the environment. Whereas the impact of environmental factors on shaping bacterial community structures (BCS) was relatively well covered, the impact of spatial factors was only recently taken in consideration (Romina Schiaffino et al., 2011). Reasons for this shortcoming in research work carried out so far arose from inadequate sampling methodologies or from lack of in-depth techniques able to explore the diversity of the BCS or even from the inherent difficulty to merge different sets of data. Following the development of fingerprinting and high-throughput techniques, an intense debate started on the question whether BCS show biogeographic signatures in their distributions or not. Fenchel and Finlay (2004) proposed that microbes (<1 mm in length) have a cosmopolitan distribution, a flat relationship between species and area, and at most a weak latitudinal gradient of diversity. An increasing number of studies is challenging the “*Everything is everywhere*” hypothesis (De Wit and Bouvier, 2006), suggesting that physical isolation may be more widespread than previously thought both in conventional and in rare ecosystems (Papke and Ward, 2004). Recent efforts invested in the study of microorganisms showed that microbial communities were not stochastically distributed on a wide scale. Discrete biogeographical patterns were locally strongly influenced by variables such as soil type and land use, whereas climate and geomorphology showed a lesser impact (Ranjard et al., 2010). Further studies suggested that the vast majority of the microorganisms are not ubiquitous, even within a given uninterrupted habitat, and that a biogeographical pattern is typical for microbial biocenoses (Martiny et al., 2006; Fuhrman, 2009). Recently the existence of endemic species among cyanobacteria and green algae (Vyverman et al., 2010) has been reported. At the habitat level, Oakley et al. (2010) demonstrated that significant biogeographical patterns can be clearly observed at relatively small spatial scales, even when there were no significant barriers to dispersal. As an extreme case, the biogeographical partitioning of a habitat was found in deep oceans, with a spectacular increase of both richness and evenness with depth (Agogué et al., 2011). van der Gucht et al. (2005) mentioned on the contrary that bacterial communities associated with lakes were not driven by regional effects, but that the observed microbial patterns could be explained primarily by regional discrepancies in environmental differences.

Distant aquifers can be considered as geographical islands composed of a heterogeneous assemblage of discrete macro- and micro-scale habitats, providing a large panel of living conditions and niches, which in turn allow for the balanced development of interrelated bacterial guilds (Goldscheider et al., 2006). Biological interactions are also able to impose a spatial zonation in apparently homogenous geological structures (Bethke et al., 2008). The consequent geographical localization of specialized bacterial guilds is all the more congruent as these habitats are isolated spatially, showing relatively slow and globally unidirectional water fluxes (Chapelle et al., 1995; McGuire et al., 2005). The spatialization and regionalization of the OHRB guild have rarely been investigated (Dowideit et al., 2010). So far, the spatial variability of bacterial communities present in CE-contaminated sites has not yet been examined and it is not known whether the geographical localization of contaminated sites could have a direct effect on OHR remediation capacities. Tas et al. (2009) have shown that spatial and temporal fluctuations affected populations of obligate OHRB guild members in river sediments, as a result of both environmental variables and long term pollutant exposition. In contrast, Krzmarzick et al. (2012) demonstrated that OHRB-*Chloroflexi* members were widely distributed in uncontaminated terrestrial ecosystems. The distribution pattern of microbial communities involved in OHR over a large geographical scale of habitats, as well as the proper identification of the environmental factors structuring their composition, is still poorly documented. Processes governing the ecology of these communities are nevertheless of great interest from a fundamental point of view as well as for the application of case-specific efficient bioremediation strategies. The objective of the present study was therefore to assess the BCS associated with geographically distant CE-contaminated aquifers and to explore the separate and combined effects of the environment, the geographical position and the CE concentrations on these communities. More specifically, the focus of this study was set on the relationship between aquifer communities and their local degradation capacity, addressing a hypothetical endemism of obligate OHRB guild members.

## Materials and Methods

### Sampling

A total of 84 aquifer groundwater samples (1000 ml) were taken manually using a submerged 12 V pump connected to a PTFE tubing and were kept in the dark at low temperature until processing (Table 1). Bacterial cells were collected by filtration onto a 0.2 μm polycarbonate sterile filter (Millipore) under a laminar flow hood so as to prevent any contamination of the samples. The filters were kept frozen (−25°C) in sterile plastic bags (Whirlpack, USA) until DNA extraction.

**Table 1:** **Details about the five CE-contaminated aquifers and their geographical locations**.

Aquifers	Location	Country	Samples #	Contaminant	Aquifer structure
A	Lyss	Switzerland	9	PCE	Quaternary deposits
B	Bulles	Switzerland	22	PCE	Quaternary deposits
C	Bitterfeld	Germany	20	PCE*	Quaternary and tertiary deposits
D	Zuchwil	Switzerland	17	PCE	Quaternary deposits
E	Lyss	Switzerland	16	PCE	Quaternary deposits

All test-fields were under Monitored Natural Attenuation (MNA). *The Bitterfeld test-field was multi-contaminated (Imfeld et al., 2011).

### Chemical analyses

Values for temperature, pH, and oxygen concentration were obtained *in situ* using calibrated field probes. Nitrate and sulfate concentrations were measured by ion chromatography. Soluble iron was measured by inductively coupled plasma atomic emission spectroscopy (ICP-AES). Chloroethene concentrations were obtained in the head space of water samples and measured using a gas chromatograph coupled with a flame ionization detector (Nijenhuis et al., 2007; Aeppli et al., 2008).

### Bacterial community analysis

DNA extraction was carried out from filters using the FastDNA SPIN kit for soil (MP Biomedicals, France) according to the manufacturer instructions and was eluted in 10 mM Tris-HCl buffer (pH 7.5). Aliquots were stored at −25°C until analysis. Samples from aquifers D and E were processed using Terminal Restriction Fragment Length Polymorphisms (T-RFLP) as described previously (Rossi et al., 2009). PCR products from DNA samples isolated from the aquifers A, B, and C were generated with a combination of HEX-labeled Eub8f and unlabeled Univ518r primers according to identical PCR conditions. One microliter of HaeIII digested amplification products was mixed with 8.5 μl of HiDi formamide (ABI) and 0.5 μl of GS500-ROX standard (ABI). Denatured samples were loaded onto an ABI 3100 DNA capillary sequencer equipped with 50 cm long capillaries and POP 6 electrophoresis matrix according to the manufacturer instructions. The resulting datasets were analyzed with Genescan (ABI).

### EndPoint PCR amplifications

Presence of 16S rRNA genes of *Dehalococcoides* spp. and *Dehalobacter* *restrictus* in the extracted DNA samples was analyzed by EndPoint PCR using respectively the primers DHC587f (3^′^-GGACTAGAGTACAGCAGGAGAAAAC-5^′^) and DHC1212r (3^′^-GGATTAGCTCCAGTTCACACTG-5^′^; Hendrickson et al., 2002), and Dre441f (3^′^-GTTAGGGAAGAACGGCATCTGT-5^′^) and Dre645r (3^′^-CCTCTCCTGTCCTCAAGCCATA-5^′^; Smits et al., 2004). Amplification reactions were conducted as follows in 10 μl reaction volume: 1 μl 10× PCR buffer (Promega, Madison, USA), 0.25 μl of both primers at 10 μM, 0.8 μl of 10 mM dNTPs, 0.5 U of GoTaq DNA polymerase (Promega, Madison, USA) and 0.1 ng/μl template DNA (final concentration), completed with sterilized and UV-treated MilliQ water (Millipore, USA). PCR amplifications were conducted as follows: initial denaturing step at 94°C (4.5 min), followed by 35 cycles of 0.5 min denaturation at 94°C, 1 min annealing at the corresponding annealing temperature, 1 min elongation at 72°C and a final elongation step of 10 min at 72°C. PCR products were examined in a 1.5% agarose gel to confirm the specificity of the amplification reaction and to confirm presence or absence of these genes.

### Statistical analysis

Numerical treatment and analysis of the data were carried out with R (http://www.r-project.org/index.html) using the vegan and the fda packages (Oksanen et al., 2009; Ramsay et al., 2009; Borcard et al., 2011). T-RFLP fingerprinting profiles of the bacterial communities were explored using a Functional Principal Component analysis (FPCA; Illîan et al., 2009). Functional data analysis methods comprise a group of statistical methods that handle mathematical functions rather than single values (Ramsey and Silverman, 2005). FPCA treats T-RFLP profiles as mathematical functions, taking specifically into account the position of each T-RF along the profile and explicitly including neighborhood structures in the analysis. It finds typical shapes of these functions and forms groups. A possible disparity in the migration pattern of the restriction fragments, due, e.g., to the use of two different capillary polymers, is thereby minimized. It was shown recently that FPCA significantly improved the detection of profiles measured through molecular fingerprinting techniques when compared to the traditional Principal Component Analysis (PCA; Illîan et al., 2009).

In order to assess the effect of the spatial scale on BCS, FPCA was applied for the 84 samples together, defining here a large geographic scale, the maximum distance between aquifer being approximately 1000 km. It was also applied to the samples of the aquifers A, D, and E defining a medium geographic scale, the average distance between these three aquifers being approximately 50 km. For the FPCA including all aquifers, the coordinates of the samples on the first ordination axis were used to test the overall aquifer effect by means of ANOVA, whereas pair-wise comparisons were made with Tukey–Kramer Honestly Significant Difference (HSD) tests. So as to minimize any possible bias induced by differences in the two bacterial community analyses (see above), statistical tests were not directly done on raw FPCA coordinates, but were carried out on the residuals of a simple linear regression with “*Methodology*” as a qualitative explanatory co-variable with two levels. Relationships between the bacterial communities and their respective environmental conditions were also tested using the coordinates of the samples on the FPCA ordination axis. In this case Pearson correlations were calculated between the environmental and pollutant variables and sample coordinates on the first or the second axis of the FPCA. As for the ANOVA, the “*Methodology*” effect was removed before the analysis of the FPCA.

Partial regressions were used for the partitioning of the influence of the geography, the chemical environment and the contaminant (CEs) on the BCS. For this analysis, we used three sets of explanatory variables: (i) “*Geography*” which encoded the geographical position of the aquifers (longitude and latitude), (ii) “*Chemistry*” which included a set of selected environmental variables such as conductivity, pH, O_2_, ${\text{SO}}_{\text{4}}^{\text{2–}}$, and Fe^2+^, and finally (iii) “*Contaminants*” which included the concentrations of the PCE, TCE, cDCE, VC as well as the total CEs. To avoid important differences between the numbers of explanatory variables within each set, only variables showing significant correlation with the Pearson Correlation Coefficients at least for one of the two first axes of the FPCA (see above) were selected in the model. The dependent variable was again the corrected coordinates of the samples on the first axis of the FPCA. Partial regressions permitted to extract the variation in the FPCA first axis explained by each of the three sets of explanatory variables and shared by these three data sets (Borcard, 2002).

Partial Mantel tests were used to examine the influence of geographical distances (straight-line distances between sampling positions) on the BCS with “*Methodology*” as a co-variable. Similarities between T-RFLP profiles were calculated using the Bray–Curtis distance metric (Legendre and Legendre, 1998), which has been recommended for T-RFLP data sets (Rees et al., 2004). For the “*Methodology*” distance matrix, 0 was attributed for couple of samples with the same “*Methodology*” and 1 for couple of samples with different ones. Like for FPCA, the analysis was done for all samples together (long geographic distances) and for the aquifers A, D, and E (medium geographic distances). Furthermore, each aquifer was also analyzed individually, defining a local geographical scale for this specific analysis.

Mantel tests were also conducted between total T-RF numbers observed for each aquifer and the size of the habitat provided by the surface of the aquifer which was accessible to sampling, the latter being restricted physically by the density of sampling wells and boreholes covering the sites.

## Results and Discussion

To date, efforts in evaluating CEs reductive dechlorination processes in aquifers and in monitoring their *in situ* remediation potential provided contrasting results (Stroo et al., 2010). Even under very similar environmental conditions, groundwater microbial communities have shown variable remediation capacities (Lorah and Voytek, 2004; Bradley and Chapelle, 2010). The accumulation of toxic intermediates is believed nowadays to result either from an incomplete sequence of dechlorination, or from a decreased efficiency in the dechlorination of lower CEs (Papke and Ward, 2004; Bombach et al., 2010). The five aquifers selected in this study, although showing clear active OHR processes, did not derogate from this rule (Aeppli et al., 2010; Imfeld et al., 2011). Aquifer A showed an overall limited degradation capacity, with the concomitant accumulation of cDCE, whereas aquifers B, C, D, and E demonstrated the capacity to degrade completely the contaminant to ethene (data not shown). However, this complete dechlorination was not attained globally in these aquifers and large variations in the degradation efficiency could be observed locally. As mentioned by Shani (2012), variations in the local terminal electron-accepting processes (TEAPs) as well as a large variability in the geological structures were responsible for strong variations in the degradation capacities of aquifers D and E respectively. Furthermore, and in both cases, the accumulation of cDCE and VC was shown to be fluctuating both in time and space, confirming other recent findings (Dowideit et al., 2010).

The presence of obligate OHRB guild members, such as *Dehalococcoides* spp. and *D. restrictus*, were examined in all aquifer samples by EndPoint PCR amplification using specific primers (Imfeld et al., 2011; Shani, 2012). These OHRB are restricted to H_2_ as the sole electron donor and to organohalide compounds as their electron acceptors. They were detected repetitively in the observed contaminated aquifers, except in the samples of the aquifer A. This finding confirmed previous studies carried out on the widespread distribution of obligate OHRB. For instance, Hendrickson et al. (2002) found that *Dehalococcoides* spp. was present in 21 contaminated sites where complete dechlorination to ethene was observed. This genus was absent at the three sites were dechlorination was not proceeding beyond cDCE, suggesting that its absence at a site may prevent complete OHR. Additionally, Krzmarzick et al. (2012) showed that *Chloroflexi*-related OHRB guild members were widely distributed in uncontaminated soil habitats. Similar results were obtained for *Dehalobacter*-related OHRB, with the successful isolation of closely affiliated sequences from dichloromethane-degrading enrichment cultures inoculated with pristine river sediment (Justicia-Leon et al., 2012). Further evidence showed that the observed diversity in obligate OHRB may be underestimated due to the use of suboptimal detection techniques. For instance, Flynn et al. (2000) showed that *Dehalococcoides* sp. was absent from enrichment cultures that dechlorinated PCE to ethene, indicating that simple molecular characterization based on this genus only may underestimate the indigenous OHR potential. Organisms related to the Lahn Cluster (Kittelmann and Friedrich, 2008) and to *Dehalogenimonas lykanthroporepellens* (Moe et al., 2009) could play an important role in the complete degradation of CEs in certain habitats, suggesting that the phylum *Chloroflexi* might contain OHRB genera and species still to be discovered (Rouzeau-Szynalski et al., 2011).

Analysis of T-RFLP data sets showed that those T-RFs, which were related theoretically to known OHRB guild members, contributed marginally to the communities of aquifers A, B, and C, even when, as a hypothesis, their respective contributions were credited totally to organohalide respirers (data not shown). Aquifers D and E presented a different pattern. T-RF 165 that was identified unambiguously as *Dehalococcoides* spp. (Rouzeau-Szynalski et al., 2011; Shani, 2012), accounted in selected samples for up to 14 and 3.5% of the total T-RFLP fluorescence, respectively. Furthermore, T-RF numbers per water sample varied significantly in the five aquifers, ranging from a maximum 133.2 ± 30.2 (aquifer A) to as low as 55.6 ± 8.4 (aquifer E), revealing large differences in the apparent community structures. Mantel tests showed equally that the numbers of T-RFs were not correlated with the size of the habitat that was accessible, the latter being restricted physically by the density of sampling wells and boreholes covering the sites (data not shown). Contrastingly Reche et al. (2005) showed that a very consistent pattern could be found between numbers of bacterial OTUs and lake areas (Sierra Nevada, Spain). Identical results were obtained by Bell et al. (2005) who reported on an increase in bacterial diversity with increasing size of tree hole habitats. In the present study, the observed variation in T-RF numbers could be attributed possibly to small-scale variations in environmental conditions. It is however not to be excluded that the reduced accessibility to the groundwater habitat could result in an apparent simplified diversity of the communities in such complex habitat structures.

**Figure 1:**
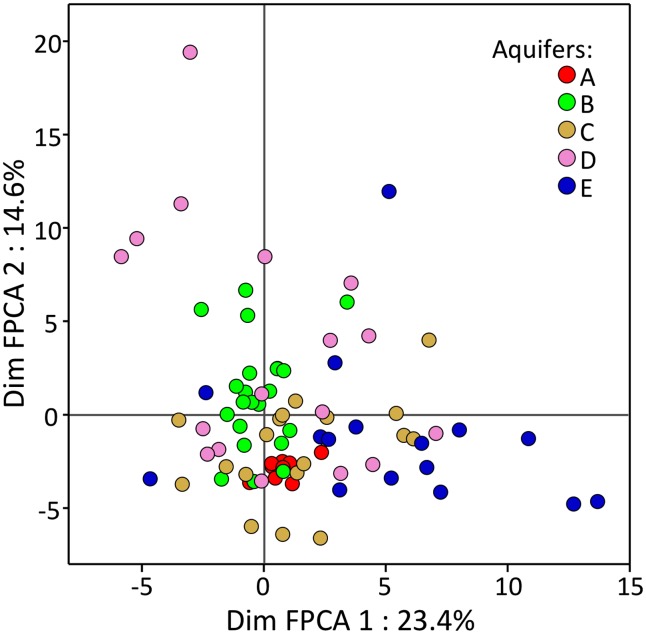
**Functional principal component analysis (axis 1 and 2) carried out on T-RFLP profiles generated from the bacterial communities present in 84 groundwater samples taken from five CE-contaminated aquifers**. Different colors are used to display samples from the different aquifers. Axis coordinates were corrected for “*Methodology*”.

**Figure 2:**
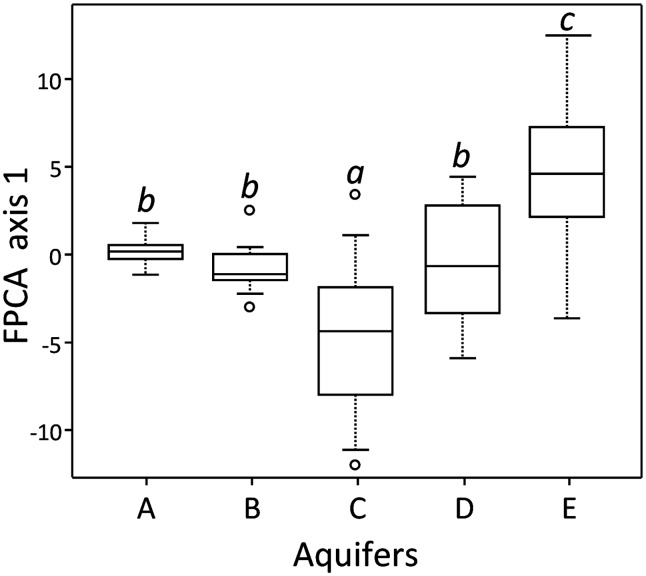
**Coordinates of records for the first ordination axis of the FPCA, corrected for “*Methodology*” and grouped by aquifers**. Significant differences (*P* ≤ 0.05) between sets of coordinates computed with the Tukey–Kramer HSD test are indicated with different small capitals.

At the community level (Figure 1), FPCA analysis on T-RFLP data revealed that the BCS were displaying singular patterns, with clusters showing very different sizes. The ANOVA on sample scores along with the first FPCA axis showed that aquifers were significantly different (*P* < 0.001). The communities present in the water samples of aquifer A formed a tight cluster, denoting comparatively homogenous BCS among all samples. In contrast, T-RFLP samples of the aquifers D and E presented a broader area on the two first axes of the FPCA ordination plot, denoting more heterogeneous BCS. Interestingly, the FPCA patterns of the five aquifers were not totally disconnected from each other and were partly overlapping, confirming that a variable proportion of T-RFs were shared among all aquifers. Significant differences among T-RF compositions could be assessed using the Tukey–Kramer HSD test. The BCS found in the aquifers C and E were characterized by a significantly unique T-RF signature, whereas aquifers A, B, and D shared significant traits (Figure 2).

Pearson correlations were computed in order to assess the effect of environmental factors on the BCS. The aquifers were grouped as a function of their geographical positions, defining two geographical average distances. The large scale was defined by the maximal distance found between two of these five aquifers (approximately 1000 km), whereas the medium scale was given by the distances between aquifers A, D, and E, which are located at an average distance of about 50 km from each other. Finally, Mantel tests and partial Mantel tests were carried out on the samples for the confirmation of these results. These tests enabled us also to evaluate the potential geographical impact at a local aquifer scale (approximately 100 m scale). As a recall, the initial withdrawal of information regarding the methodological aspect of T-RFLP analysis (co-variable “*Methodology*”) also induced the loss of information regarding the origin of all samples.

Water samples taken from three aquifers (A, D, and E), located geographically at a maximal distance of about 50 km, revealed significant correlations with environmental variables composed of electron acceptors, such as oxygen, nitrate, and sulfate (Table 2). Fe^2+^, the product of dissimilatory iron-reduction, was the only variable showing significant negative correlation on the second axis of the FPCA. The significant correlations of BCS with TEAPs demonstrated the strong structuring of the communities around key redox reactions, indicating thereby also the presence of a possible competitive mechanism between obligate hydrogenotrophic OHRB with other anaerobic respiring bacteria, such as nitrate-, iron(III)-, and sulfate-reducers for electron donors (Bradley and Chapelle, 2010). Interestingly, no significant correlation was obtained at this scale between BCS and CEs, indicating either that each aquifer community responded in a specific way to the presence of the contaminant or that a possible common response had little or no impact on their structuring. Concordant evidence (see for instance Bradley and Chapelle, 2010) suggested that reductive dechlorination of CEs is attributable to cooperative microbial consortia structured around OHRB rather than to the activity of single OHRB populations. Another explanation could arise from the fact that the first steps in the reduction of CEs could be carried out by facultative OHRB showing significant metabolic redundancies (e.g., *Desulfitobacterium* sp., *Geobacter lovleyi*, *Sulfurospirillum* sp., *Desulfuromonas* sp.) and for which CEs represent a single electron acceptor among the panel they can utilize.

**Table 2:** **Pearson correlation coefficient calculated between environmental variables and sets of sample coordinates on the first two axes of the FPCA, corrected for “*Methodology***.”

	Medium scale (50 km)				Large scale (1000 km)			
	Aquifers A, D, E (*n* = 42)				All aquifers (*n* = 84)			
	Axis 1 (26.3%)		Axis 2 (20.9%)		Axis 1 (23.4%)		Axis 2 (14.6%)	
	Rho	*P*	Rho	*P*	Rho	*P*	Rho	*P*
**GEOGRAPHY**								
Latitude	0.49	***	−0.18	ns	−0.51	***	−0.26	*
Longitude	0.48	**	−0.17	ns	−0.52	***	−0.25	*
**ENVIRONMENT**								
Temperature	−0.29	ns	0.19	ns	0.35	**	−0.11	ns
Conductivity	0.55	***	−0.19	ns	−0.55	***	−0.23	*
pH	−0.37	*	0.19	ns	0.50	***	0.05	ns
O_2_	−0.35	*	−0.08	ns	0.14	Ns	0.01	ns
${\text{NO}}_{\text{3}}^{\text{–}}$	−0.45	**	0.07	ns	0.07	Ns	−0.09	ns
${\text{SO}}_{\text{4}}^{\text{2–}}$	−0.46	**	0.17	ns	−0.35	**	−0.16	ns
Fe^2+^	0.59	***	−0.39	*	−0.19	Ns	0.24	*
**CONTAMINANTS**								
PCE	0.10	ns	0.23	ns	−0.20	Ns	−0.11	ns
TCE	0.11	ns	0.27	ns	−0.38	***	−0.24	*
cDCE	−0.24	ns	0.22	ns	−0.19	Ns	−0.22	*
VC	−0.12	ns	0.15	ns	−0.32	**	−0.19	ns
Total CEs	0.02	ns	0.30	ns	−0.33	**	−0.24	*

Medium scale: samples from the aquifers A, D, and E were analyzed together (*n* = 42). Large scale: all samples from the five aquifers were analyzed together (*n* = 84). *P < 0.05; **P < 0.01; ***P < 0.001. ns, statistically not significant.

PCE, and more generally CEs, were often considered from an anthropogenic point of view as an external disturbance to the aquifer systems. From an ecological point of view, they merely represent an additional TEAP for facultative OHRB such as the ones mentioned above, that will be used, or not, depending on the competitive advantage that these compounds would provide locally over other TEAPs. A significant correlation between BCS and CEs was found when all five aquifers were taken into consideration (Table 2). With the exception of PCE and cDCE, CEs showed a strong correlation on the first axis of the FPCA, and total CEs, TCE, and cDCE were correlated with the second axis of the analysis. As mentioned above, absence of correlation with PCE could be interpreted as the possible outcome of the degradation of this compound by a high diversity of potential degraders sharing the same niche in the different habitats. Significant correlations were possibly the result of a selection pressure induced by the contaminant, encouraging the development of OHRB guild members among highly divergent bacterial biocenoses. The development of these members, reaching locally a large proportion of the total T-RFs as a response to the presence of the contaminant, strengthened the impact of local environmental conditions when all samples were taken into consideration. Concomitantly, other TEAPs, such as oxygen and nitrate lost their predominant role in the structuring of bacterial communities.

The widespread distribution of obligate OHRB in geographically and physically isolated aquifer habitats poses the intriguing question of their origins as well as their hypothetical maintenance in the corresponding pristine ecosystems (Bradley and Chapelle, 2010). Part of the response doubtlessly was provided by recent advances in the analysis of halogenated hydrocarbons which are produced naturally by biotic or abiotic processes, such as volcanic activities (for a comprehensive review see Gribble, 1996, 2003). Organohalides, and more specifically CEs even if they compose only a small subset of this class of molecules, are thus occurring naturally in a wide variety of environments. Consequently, the accidental release of organohalides in the aquifer habitat is likely to cause local enrichment of OHRB that were already present. Concomitantly, the extensive distribution of OHRB guild members able to conduct the full degradation of these products is probably the reason for which such recalcitrant molecules were not found to accumulate in these habitats (Bradley and Chapelle, 2010). Our results suggested that BCS were structured to certain extent by a niche specialization dedicated to the degradation of the contaminant. As a recall, these guild members have been shown recently to be widely distributed in uncontaminated terrestrial ecosystems (Krzmarzick et al., 2012) and similar behavior could be expected for groundwater systems. In addition, the multitude of microhabitats in the aquifers probably also promotes the development of redundant niches. In these conditions, the geographical distance between aquifers would stimulate locally the development of OHRB guilds that could have evolved according to allopatric or sympatric speciation toward highly specialized actors (Fenchel and Finlay, 2006; Allison and Martiny, 2008; Oakley et al., 2010) covering new functions, while keeping an identical ribosomal signature. The resulting apparent redundancy of catabolic OHR genes present in obligate OHRB (Hölscher et al., 2004; Holmes et al., 2006) would then be considered as a natural adaptive response toward multiple patterns of environmental conditions fluctuating in time and space (Prosser et al., 2007).

Temperature and pH were either not at all or only weakly correlated at medium geographical scale with BCS (Fierer and Jackson, 2006). Both factors increased their correlation at a large geographical scale, which confirmed preceding findings carried out on soil bacterial communities. Ayuso et al. (2009) presented temperature as the main factor affecting the spatiotemporal distribution of microbial communities in a sandy coastal aquifer system. Similarly, Bryant et al. (2008) found that bacterial taxon richness and phylogenetic diversity decreased monotonically from the lowest to the highest geographical elevations, indicating a strong impact of temperature on bacterial communities. In our study, results of the variance analysis further demonstrated that the observed bacterial communities were structured with an almost identical contribution by both the localization of the aquifer and the local environmental conditions, confirming other observations carried out in lake ecosystems (Romina Schiaffino et al., 2011). In this study, the chlorinated contaminants contributed to a lesser extent at the medium scale and became important at a large geographical scale, when all aquifers were considered together.

Two statistical analyses carried out in this study, namely the Pearson Correlation Coefficient and the Mantel test, demonstrated the strong impact of geography on the structuring of the bacterial communities (Tables 2 and 3). Results of the first test, at the medium geographical scale, showed that the aquifers A, D, and E correlated significantly with both latitude and longitude. At large geographical scale, this correlation became significant also for the second axis of the analysis. Contrastingly, Mantel tests carried out at the local scale (at the scale of each aquifer) did not reveal any correlation with the spatial position of the samples within each aquifer, a possible indication showing that the contaminants had little or no impact on the structuring of the communities. The similarity in community composition between sites decreased with increasing geographic distances. The correlation was *r* = 0.193 (*P* < 0.001^***^) when BCS of three aquifers located at medium geographical range were analyzed and slightly increased to *r* = 0.223 (*P* = 0.001^***^) when long geographical range was taken in consideration. Latitudinal gradients have already been found in different microbial habitats. For instance, Fuhrman (2009) mentioned the presence of strong patterns of diversity in planktonic marine bacteria when analyzing samples from tropical to polar latitudes. Romina Schiaffino et al. (2011) found likewise that bacterioplankton communities were shaped by a combination of both spatial (latitude and longitude) and environmental factors. More specifically, Haack et al. (2004) demonstrated that spatial changes in an aquifer contaminated by fire-training activities were likely to influence the composition of the bacterial communities. In this case also, geochemistry was as important as contaminant or TEAP in discriminant analysis of community groups. Furthermore, Martiny et al. (2006) showed that environmental factors gradually ceded their relative importance in favor of increasing geographical impact as a function of the distance of the compared habitats, which appeared to be the case in the present study, with the loss of significant correlation with oxygen and nitrate with increasing distances.

**Table 3:** **Mantel test between species data sets (Bray–Curtis) and spatial data sets (Euclidean distances)**.

Aquifers (sample numbers)	Mantel statistic *r*	*P* value (999 permutations)	
A (*n* = 9)	−0.181	0.752	ns
B (*n* = 22)	−0.048	0.615	ns
C (*n* = 20)	0.200	0.085	ns
D (*n* = 17)	0.013	0.45	ns
E (*n* = 16)	−0.011	0.511	ns
ADE (*n* = 42)	0.193	0.001	***
All samples (*n* = 84)	0.223	0.001	***

For aquifers A, D, and E as well as for all aquifers, “*Methodology*” was included as a co-variable (partial Mantel test). *P < 0.05; **P < 0.01; ***P < 0.001. ns, statistically not significant.

**Figure 3:**
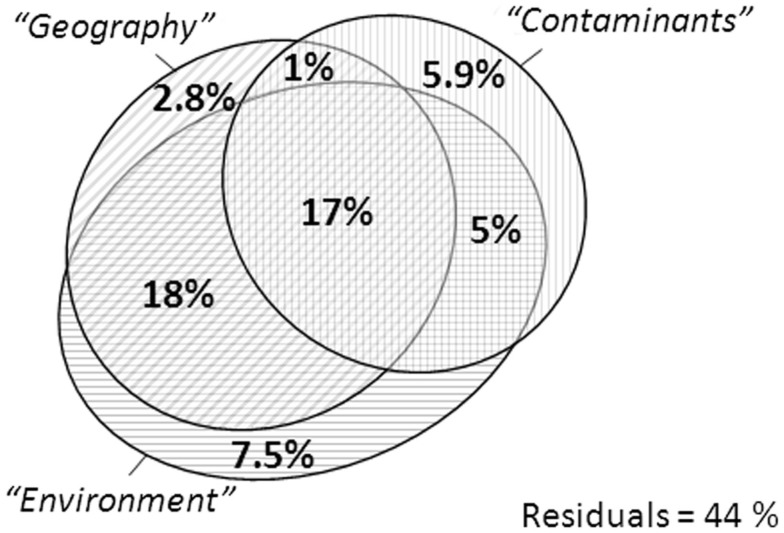
**Variance partitioning on the first axis of the above mentioned FPCA carried out on 84 samples and corrected for “*Methodology*.”** “*Geography*,” “*Environment*” and “*Contaminants*” groups of explanatory variables include only those variables which showed a significant effect in Table 1.

The variation on the first axis of the FPCA could be explained to a large extent (56%) by a combination of three sets of explanatory variables (Figure 3). Although a significant amount of variance in the system could be identified, large amount of biotic and abiotic variations still remain unexplained. Possible impact induced by a restricted accessibility to the habitat or unmeasured variability may in part contribute to the unexplained variation. Interestingly, shared variance between geography and environmental factors were almost completely overlapping. “*Geography*” alone accounted only for 2.8% of the variance of the system. The overlap strongly confirmed that the habitats were spatially structured. On the other hand, “*Geography*” alone contributed marginally to the structuring of the BCS, and the apparent significant correlations that were computed beforehand were most possibly induced indirectly via regional environmental differences confounded in spatial variations. The variance expressed by the “*Contaminants*” reached a total of 28.9%, from which a major proportion was confounded with both “*Geography*” and “*Environment*.” Alone, this group of explanatory variables expressed 5.9% of the variance, about the double of the variance expressed by “*Geography*” alone. These numbers confirmed the results obtained above with Pearson correlations, suggesting that the bacterial communities were structured to a certain extent by a niche specialization dedicated to the degradation of CEs. From another point of view, the presence of the contaminant likely induced a selection pressure on whole BCS due to the inherent toxicity of the compounds.

The observed differentiation in BCS among geographic regions that was strongest between aquifers located at a distance of about 1000 km could be explained by several factors. As stated by van der Gucht et al. (2007), rather than every species potentially being everywhere, the array of taxa that may colonize a given area could be restricted to more regional taxon pools. Alternatively, the patterns observed in our study may reflect regional differences in environmental conditions, either not measured or confounded in spatial differences. Several studies attributed the high degree of spatial variability in microbial activity and abundance in subsurface sediments to differences in grain size or pore size between strata (Krumholz, 2000; Goldscheider et al., 2006; Musslewhite et al., 2007). Contrastingly, increasing the distances between aquifers also increased the correlations with selected CEs, especially with low chlorinated ones, indicating that a subset of the bacterial communities was found recurrently.

The original niche in which obligate OHRB specialized offers little alternative against drastic changes in their surrounding environmental conditions. A possible adaptive response, and the subsequent survival of the cells, is likely to be the large number of the catabolic reductive dehalogenase genes (Hölscher et al., 2004; Tas et al., 2010), and a rapid evolution thereof, targeting a large panel of halogenated organic molecules. This adaptive mechanism could explain the multiplicity and the apparent redundancy of these genes present in different strains of *Dehalococcoides* spp. As a result, the successful elimination of organohalide contaminants would be accompanied by a rapid development of specialized bacterial populations, which would arise apparently from nowhere using traditional detection techniques. The existence of obligate OHRB guild members in the vast majority of organohalide-contaminated or pristine aquifers provided additional evidence that these organisms are widespread in the environment (Bradley, 2003). In a more general way, the presence of obligate OHRB in both pristine and contaminated aquifers raises the question of the endemism of microorganisms exhibiting very specialized metabolic activities in environmental systems.

## Conflict of Interest Statement

The authors declare that the research was conducted in the absence of any commercial or financial relationships that could be construed as a potential conflict of interest.
